# Xanthohumol Inhibited Mechanical Stimulation-Induced Articular ECM Degradation by Mediating lncRNA GAS5/miR-27a Axis

**DOI:** 10.3389/fphar.2021.737552

**Published:** 2021-09-10

**Authors:** Tiansheng Zheng, Qingluo Zhou, Jishang Huang, Jinliang Lai, Guanglin Ji, Dechao Kong

**Affiliations:** ^1^ Department of Orthopedics, The First Affiliated Hospital of Gannan Medical University, Ganzhou, China; ^2^ Department of Emergency, The First Affiliated Hospital of Gannan Medical University, Ganzhou, China; ^3^ Department of Trauma Center, Shanghai General Hospital, Shanghai Jiao Tong University School of Medicine, Shanghai, China

**Keywords:** osteoarthritis, extracellular matrix, mmp-13, xanthohumol, Gas5, miR-27a

## Abstract

Osteoarthritis (OA) is histopathologically marked by extracellular matrix (ECM) degradation in joint cartilage. Abnormal mechanical stimulation on joint cartilage may result in ECM degeneration and OA development. Matrix metalloproteinase 13 (MMP-13) is one of the catabolic enzymes contributing to the degradation of ECM, and it has become the potential biomarker for the therapeutic management of OA. Xanthohumol (XH), a naturally occurring prenylflavonoid derived from hops and beer, shows the protective activity against OA development. However, the potential mechanisms still need great effort. In this article, mechanical stimulation could significantly increase the expression of MMP-13 and lncRNA GAS5 (GAS5) and promoting ECM degradation. These could be effectively reversed by XH administration. Suppressed expression GAS5 ameliorated mechanical stimulation-induced MMP-13 expression. MiR-27a was predicted and verified as a target of GAS5, and overexpression of miR-27a down regulated the expression of MMP-13. Collectively, XH exhibited protective effects against mechanical stimulation-induced ECM degradation by mediating the GAS5/miR-27a signaling pathway in OA chondrocytes.

## Introduction

Osteoarthritis (OA) is histopathologically marked by progressive degradation of extracellular matrix (ECM) in joint articular cartilage. Mechanical stimulation, implicated in articular cartilage development and maintenance, is dynamic, and it is supposed to support the synthesis of extracellular matrix (ECM) under normal mechanical loading and inhibit the production of ECM under persistent improper loading (Fang et al., 2021). Many studies have reported that exercise affects the physiological actions of a joint in a mechanical intensity-dependent manner. Moderate intensity of exercise plays a critical role in the prevention and therapeutic management of OA, whereas excessive intensity is assumed to induce or exaggerate OA development (Ni et al., 2013; Song et al., 2021). The chondrocyte is the unique cell type in the articular cartilage, and the biochemical activities of chondrocytes responding to mechanical stimulation might affect the biological components and functions of ECM. Excessive mechanical stimulation has been demonstrated to be associated with increased chondrocytes apoptosis in OA cartilage (Yang et al., 2020). However, the relationship between mechanical stimulation and ECM degradation is still under investigation.

Long noncoding RNA (lncRNA), more than 200 nucleotides, usually cannot be translated into proteins due to lack of an open reading frame (Andrey and Duboule, 2014). Many dysregulated lncRNAs have been involved in the pathological changes of OA, including ECM degradation (He et al., 2021). LncRNA H19 has been demonstrated to ameliorate the mechanical force-induced cartilage degeneration in developmental dysplasia of the hip by mediating miR-485/Dusp5 axis, as indicated by increased expression of COL2A1 and aggrecan and decreased expression of MMP3 and ADAMTS5 in intermittent cyclic mechanical stress (ICMS)-induced chondrocytes (Wang et al., 2020). LncRNA MSR has been reported to be enhanced in response to mechanical stress in chondrocytes. Up regulation of lncRNA MSR facilitates pathological development and leads to cartilage degradation (Liu et al., 2016). Growth arrest specificity 5 (GAS5) is involved in the pathological mediation of OA. Increased expression of GAS5 was observed in human OA cartilage tissues. Forced expression of GAS5 increases the apoptosis rate and inhibits the proliferative activity by sponging miR-137 in chondrocytes (Gao et al., 2020). In contrast, silencing GAS5 expression results in decreased apoptosis and enhanced proliferation in OA chondrocytes (Ji et al., 2020). However, the roles of GAS5 in mechanical stimulation-dependent OA are still unclear.

Xanthohumol (XH) is a naturally occurring prenyl-flavonoid extracted from hops and beer. It has been shown that XH effectively prevents the over-production of hyaluronan, protecting against the early development of OA (Stracke et al., 2011). Specifically, XH can activate NRF2/HO-1 and inhibit NF-κB pathways, reducing cisplatin-induced nephrotoxicity (Li et al., 2018). Our previous study showed that XH could protect OA chondrocytes against IL-1β-induced inflammation and ECM degradation by activating NRF2/HO-1 and inhibiting NF-κB pathways (Zhang et al., 2021). However, the effects of XH on mechanical stimulation-induced OA chondrocytes are still unclear.

## Materials and Methods

### General

This project (GMU201809038) was approved scientifically by the Institutional Animal Care and Use Committee of the First Affiliated Hospital of Gannan Medical University and carried out under the guideline of Animal Care and Use.

### Duplication of Mice Osteoarthritis Models by Forced Running

Eight-week-old male C57BL/6 mice were obtained from the Animal Center of Gannan Medical University (Ganzhou, China). Mice were divided into four groups: 1) the negative control group (non-running), 2) the model group (running), 3) the low-dose group (running + treatment with XH (purity≧98%, dissolved in 0.5% carboxymethyl cellulose (CMC) by intragastric administration) at the dose of 30 mg/kg/d (Miranda et al., 2016)), and 4) the high-dose group (running + XH (60 mg/kg/d (Miranda et al., 2016))). Mice were subjected to forced running on a treadmill. The running protocols (Yamagishi et al., 2018) were designed with minor modifications. Simply, mice were forced to run at the speed of 30 m/min for 30 min/day on 5 days/week. The running distance by each mouse was 36 km after 8 weeks. The knee joints were collected and kept in a −80°C refrigerator for gross observation, histochemical examination, and immuno-histochemical evaluation.

### Cell Isolation and Culture

The articular cartilage from the terminal femur and upper tibia in the knee joints was collected under sterile conditions. The cartilage was then cut into small pieces and digested with pancreatic enzyme (0.25%) and collagenase II (0.2%) at 37°C for 4 h. The collected cells were cultured in DMEM (low glucose) supplemented with 10% FBS and penicillin/streptomycin in an incubator with 5% CO_2_ at 37°C. The second or third generation of the isolated chondrocytes was used for the following experiments.

### Cyclic Mechanical Stimulation Application

Cells (1×10^5^ cells/well) cultured on the 6-well BioFlex plates coated with collagen type I (Flexcell Int. Co., Hillsborough, NC, United States) at 80% confluence were transferred to a computer-controlled Flexcell Tension System (FX-4000) (Flexcell) with 20% surface elongation at a frequency of 6 cycles/min. Cells were harvested after 24 h (Xu et al., 2019).

### Cell Viability Assays

The chondrocytes viability was determined using the CCK-8 kit. Simply, cells (5,000 cells/well) were grown in 96-well plates for 24 h. CCK-8 solution (10 μL) was added carefully and co-incubated at 37°C for 4 h. The 450 nm wavelength for absorbance was used for detection using a microplate system.

### Cell Transfection

The shRNAs GAS5 (5′-ACT​TGC​CTG​GAC​CAG​CTT​AAT-3′) and a scrambled shRNA control (sh-NC: 5′-GCC​CAC​TTA​CAC​TTG​AGC​A-3′) were obtained from GeneChem (Shanghai, China). They were inserted into pGPH1/Neo and then transfected into chondrocytes using lipofectamine 3000 (Invitrogen), according to the procedures of the manufacturer’s instructions. Neomycin was used to select the stable transfectants.

MiR-27a mimics (5′-CCA​CCA​UCG​GUU​UAG​CGU​AUG-3′), miR-27a inhibitors (5′-GGU​UUA​CCU​UCC​CGG​GAC​AAU-3′), and microRNA negative controls (miR-NC: 5′-GUG​AGG​ACC​ACC​UGU​CUC​AUU-3′) were purchased from RiboBio (Guangzhou, China). Chondrocytes at the confluence of 60% (1×10^5^ cells/well) were conducted for transfection using lipofectamine 3000, after they were cultured for 1 day in the 6-well plates. The designed concentrations of miR-27a mimics, miR-27a inhibitors, and miR-NC in the final transfection system were all 50 nM.

### Immunofluorescence

For *in situ* assays, the samples were fixed with 4% paraformaldehyde at room temperature for 48 h. Decalcification was conducted in the solution consisting of EDTA and ammonium hydroxide for 1 week. Paraffin-embedded 5-µm sections of tissues were deparaffinized in xylene and hydrated in a decreasing series of ethanol solutions. The sections were probed with the primary MMP-13 antibody in a humidified chamber at 4°C overnight and secondary horseradish peroxidase (HRP)-conjugated antibodies at room temperature for 1 h. A confocal laser scanning microscope was employed and the fluorescence intensity was detected by using ImageJ.

Chondrocytes were grown and fixed with 4% paraformaldehyde on glass coverslips. Triton X-100 (0.1%) was employed to infiltrate cell and nuclear membranes. Cells were blocked by 5% bovine serum albumin (BSA) (protease-free) and then co-incubated with the primary antibody at 4°C overnight. After that, the secondary goat anti-rabbit IgG antibody was co-incubated for 1 h at room temperature. Subsequently, the medium containing DAPI was used. A confocal laser scanning microscope was employed and the fluorescence intensity was detected by using ImageJ.

### qRT-PCR

Cartilage tissues were collected, minced, and homogenized in Trizol reagent (Invitrogen; Thermo Fisher Scientific, Inc.), according to the manufacturer’s instructions. Specifically, cartilage was homogenized in 1 ml of TRIzol reagent per 50 mg of tissue. The resultant samples in TRIzol were then mixed thoroughly with 0.2 ml chloroform (Sigma), spun down and the upper fraction was harvested. The fraction was mixed with the same volume of isopropanol, and centrifuged at 10,000 × g for 10 min at 4°C. The pellet was washed with 0.8 ml of 75% ethanol, and dissolved in RNase-free water (Ambion, Thermo Fisher Scientific, United States). All steps were performed under RNase-free conditions. The total amount of the collected RNA was evaluated based on optical density at 260 nm, with its purity determined using the 260/280 nm absorption ratio (NanoDrop 2000 spectrophotometer, Thermo-Fischer Scientific, Waltham, MA, United States).

RNA (2 μg) was reverse transcribed to cDNA using M-MLV. Quantitative PCR assays were conducted on Power SYBRs Green PCR Master Mix (Applied Biosystems, CA, United States) to detect the expression of GAS5 and MMP-13. The expression of miR-27a was detected using the Taqman MicroRNA Reverse Transcription Kit and Taqman Universal Master Mix II kit (Applied Biosystems). GAPDH and U6 were used as the internal reference for mRNA and miRNA, respectively. All the primers were obtained from Biomics. The listed primer sequences are showed as follows: GAS5 forward: 5′-CTT​GCC​TGG​ACC​AGC​TTA​AT-3′, reverse: 5′-CAA​GCC​GAC​TCT​CCA​TAC​CT-3’; MMP-13 forward: 5′-CAA​GGC​TGG​TTA​CCC​AAC​AG-3′, reverse: 5′-CAC​CTG​GGA​CAA​CTG​GAA TC-3’; GAPDH forward: 5′-AGG​TGA​AGG​TAG​GAG​TCA​ACG-3′, reverse: 5′-AGG​GGT​CAC​TGA​TGG​CAA​CA-3’; miR-27a forward: 5′-TTC​ACA​GTG​GCT​AAG​TTC​CGC-3′, reverse: 5′-CTC​TAC​AGC​TAT​ATT​GCC​AGC​CAC-3’; U6 forward: 5′-CTC​GCT​TCA​GCA​GCA​CA-3′, reverse: 5′-AAC​GCT​GCA​CGA​ATT​TGC​GT-3’. Fold changes by using 2^-△△CT^ method were indicated for the expression of miRNA and mRNA.

### Western Blot Assays

The total proteins in cells were extracted and the protein concentrations were determined by BCA protein assay kit (Beyotime). 30 μg of the total proteins were applied to 10% SDS-PAGE and then transferred onto PVDF membranes. After being blocked in TBS containing 5% non-fat milk for 1 h, the membranes were co-incubated with the primary antibodies at 4°C overnight against MMP-13 (1:1,000 dilutions, Cell signaling technology, Cat. no.69926), Col2a1 (1:1,000 dilutions, Sigma, Cat. no.SAB4500366), and GAPDH (1:1,000 dilutions, Cell signaling technology, Cat. no.5174), and then with the secondary antibody conjugated with peroxidase (1:2000 dilutions, Sigma-Aldrich, Cat. no. AP510). Protein bands were detected using the enhanced chemiluminescence detection system.

### Dual-Luciferase Reporter Assay

The predicted system StarBase v2.0 was used for screening the potential miRNA targets of GAS5. The recombinant luciferase plasmids were constructed by cloning the sequences of wild-type (WT) GAS5 into the pGL-3 luciferase basic vector. In addition, the mutant-type with its mutant binding sites for miR-27a was also constructed as MUT-GAS5. Each constructed plasmid was transfected into chondrocytes with miR-27a mimics/miR-NC using lipofectamine 3000. Following incubation for 48 h at 37°C, the activities of firefly and Renilla luciferase were detected using the Glomax 96 luminometer. Firefly luciferase reporter was quantitatively normalized to Renilla luciferase activity.

### RIP Assays

This assay was used for investigating the direct interaction between GAS5 and miR-27a by employing Magna RNA immunoprecipitation kit (Millipore). Chondrocytes (about 2×10^7^ cells) were lysed and then co-incubated with magnetic beads, which were previously coated with antibodies against Argonaute2 (Ago2, Cell Signaling Technology, Cat. no.2897). Anti-IgG was used as the negative control. The RNA expression was detected by qRT-PCR. Finally, the levels of GAS5 and miR-27a in anti-IgG and anti-Ago2 groups were compared.

### Statistical Analysis

All experiments were performed three independent times and data are expressed as the mean ± standard deviation. SPSS 20.0 software was used for statistical analysis. One-way ANOVA and Tukey’s post hoc test were used to analyze the differences between multiple groups. *p* < 0.05 was indicated a significant difference statistically.

## Results

### XH Protected Against Mechanical Stimulation-Induced Extracellular Matrix Degradation in Mice

**FIGURE 1:**
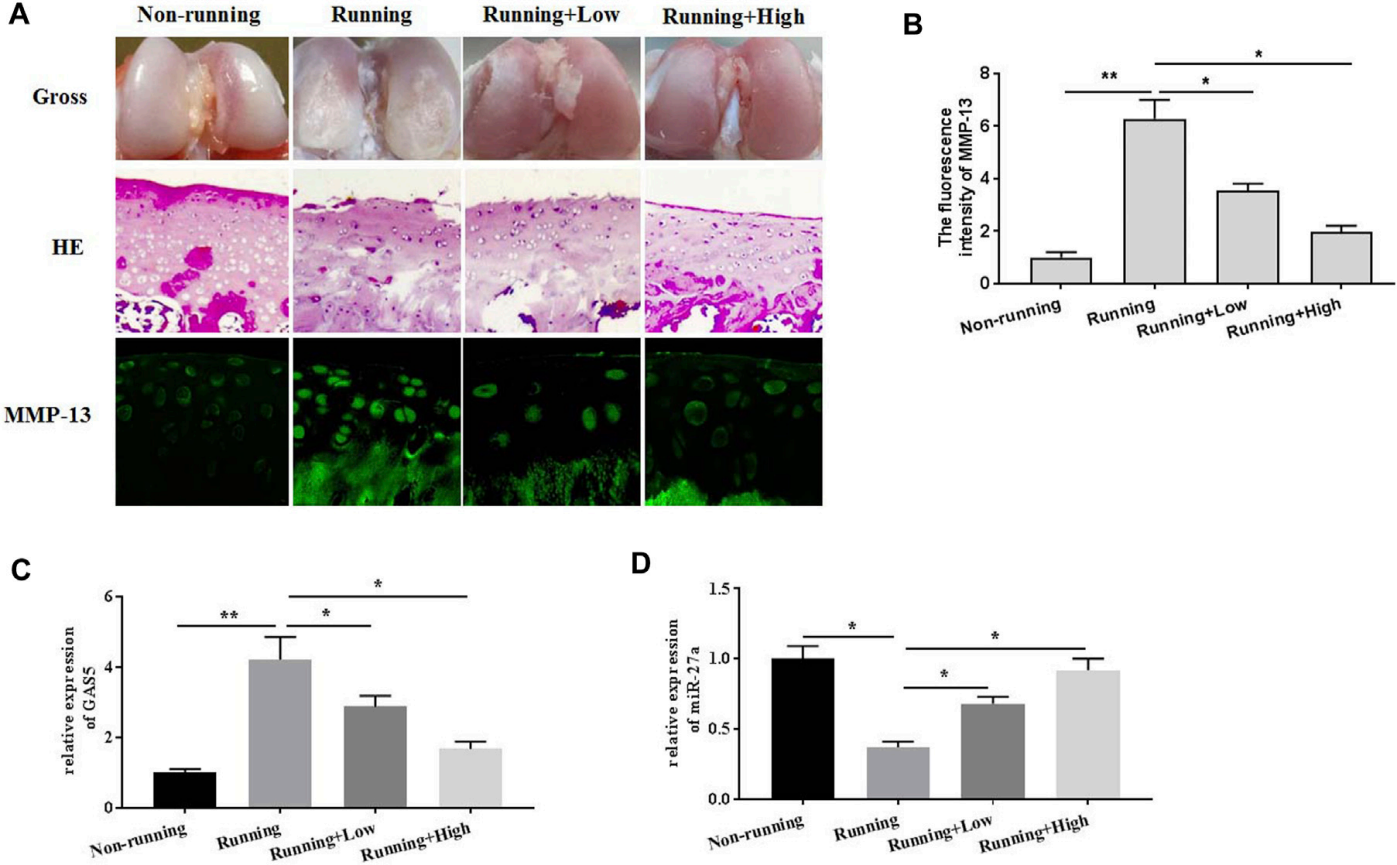
The effects of XH on mechanical stimulation-induced OA cartilage in mice were investigated. **(A)** The gross observation, histochemical examination, and immunofluorescence activity of MMP-13 were indicated (magnification×40). **(B)** The calculation of fluorescence activity of MMP-13. The expression of GAS5 **(C)** and miR-27a **(D)** in the knee joint cartilages were detected by qRT-PCR. ^*^ *p* < 0.05 and **p < 0.01. Low, the group treated with XH (30 mg/kg/d); High, the group treated with XH (60 mg/kg/d).

To explore the roles of XH on mechanical stimulation-induced OA, a forced running protocol was designed. After 8-weeks running and treatment with XH (Low, 30 mg/kg/d; High, 60 mg/kg/d) by oral administration, the mice were sacrificed. As results showed in Figure 1A, no obvious tissue damages were observed in the non-running (negative control) group in gross observation and HE staining. In contrast, the erosion on the rough surface in the cartilage of the running group was significantly showed. The immunofluorescence activity of MMP-13 (Figure 1A-B) in the running group was also dramatically increased, compared with that in the non-running group. Interestingly, administration of XH orally could effectively protect articular cartilage against mechanical stimulation-induced damage, as shown by smoother cartilage surface and thicker cartilage. In addition, XH also significantly decreased the immunofluorescence activity of MMP-13. These suggested that XH exhibited protective activity against mechanical stimulation-induced damage in mice. The expression of GAS5 (Figure 1C) and miR-27a (Figure 1D) were also determined. The results had shown that mechanical stimulation upregulated GAS5 expression but downregulated miR-27a expression. XH could reverse the effects of mechanical stimulation on cartilage.

### XH Ameliorated Mechanical Stimulation-Induced MMP-13 and GAS5 Expression

**FIGURE 2:**
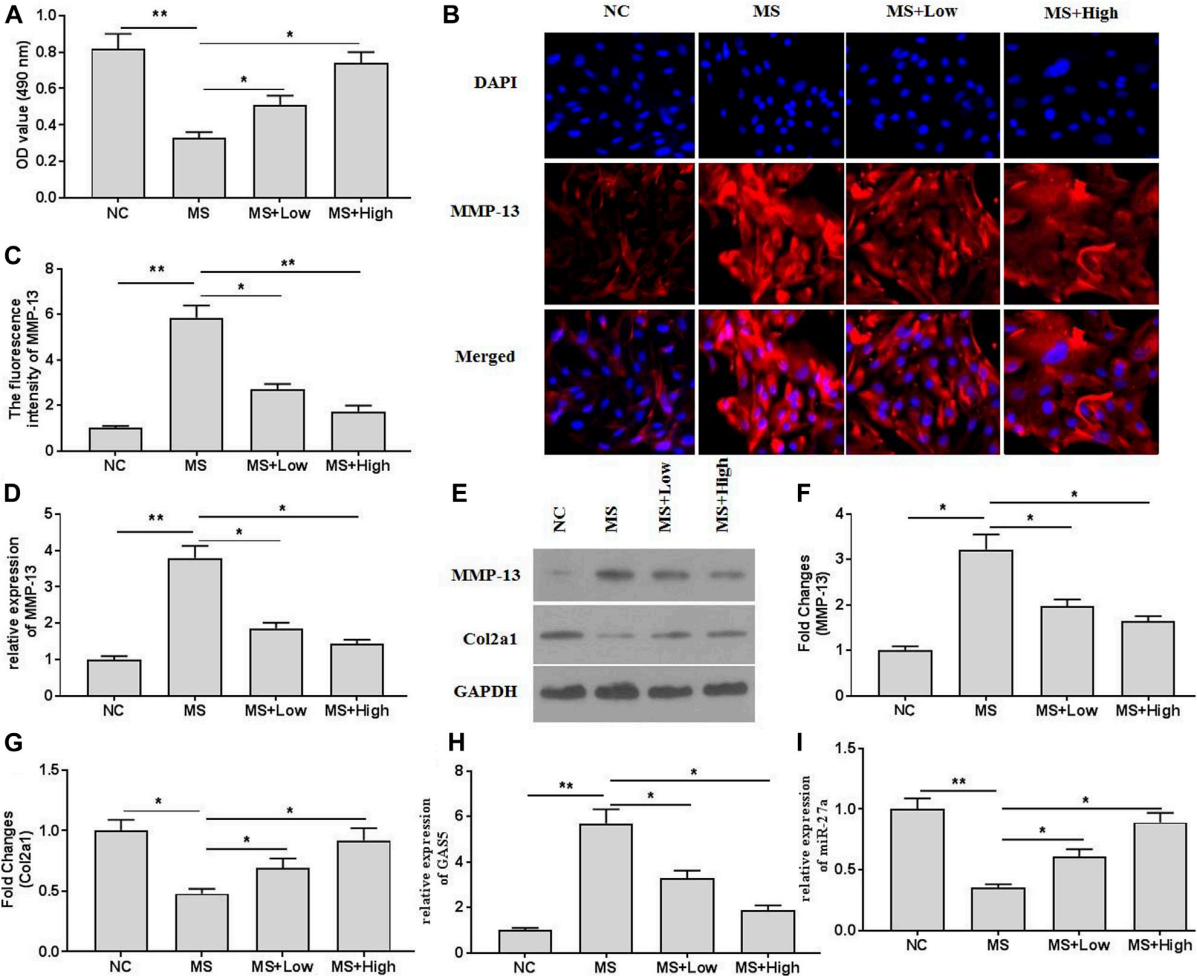
The effects of XH on mechanical stimulation-induced MMP-13 and GAS5/miR-27a expression in chondrocytes were investigated. **(A)** The cell viability was determined by CCK-8 assays. **(B)** The immunofluorescence intensity of MMP-13 in mechanical stimulation-treated chondrocytes was detected. **(C)** The calculation of fluorescence activity of MMP-13. The mRNA **(D)** expression of MMP-13 was determined by qRT-PCR, and the protein expression of MMP-13 **(E-F)** and Col2a1 **(E**, and **G)** were determined by western blotting assays. The expression of GAS5 **(H)** and miR-27a **(I)** was detected by qRT-PCR. ^*^ *p* < 0.05 and **p < 0.01. NC, negative control; MS, mechanical stimulation; Low, XH (5 μM); High, XH (20 μM).

The cell viability assays indicated that mechanical stimulation (20% surface elongation at a frequency of 6 cycles/min) could decrease chondrocytes viability (Figure 2A). XH protected chondrocytes and increased viability. The immunofluorescence assays (Figures 2B,C) also indicated that XH significantly decreased the intensity of MMP-13, compared with that in the running group. Mechanical stimulation increased the mRNA (Figure 2D) and protein (Figures 2E,F) expression of MMP-13 and decreased the expression of Col2a1 (Figures 2E,G). In addition, mechanical stimulation up regulated GAS5 expression (Figure 2H) but down regulated miR-27a expression (Figure 2I) in cultured chondrocytes. These effects could be ameliorated by XH administration.

### Suppressed Expression of GAS5 Attenuated Mechanical Stimulation-Induced MMP-13 Expression

**FIGURE 3:**
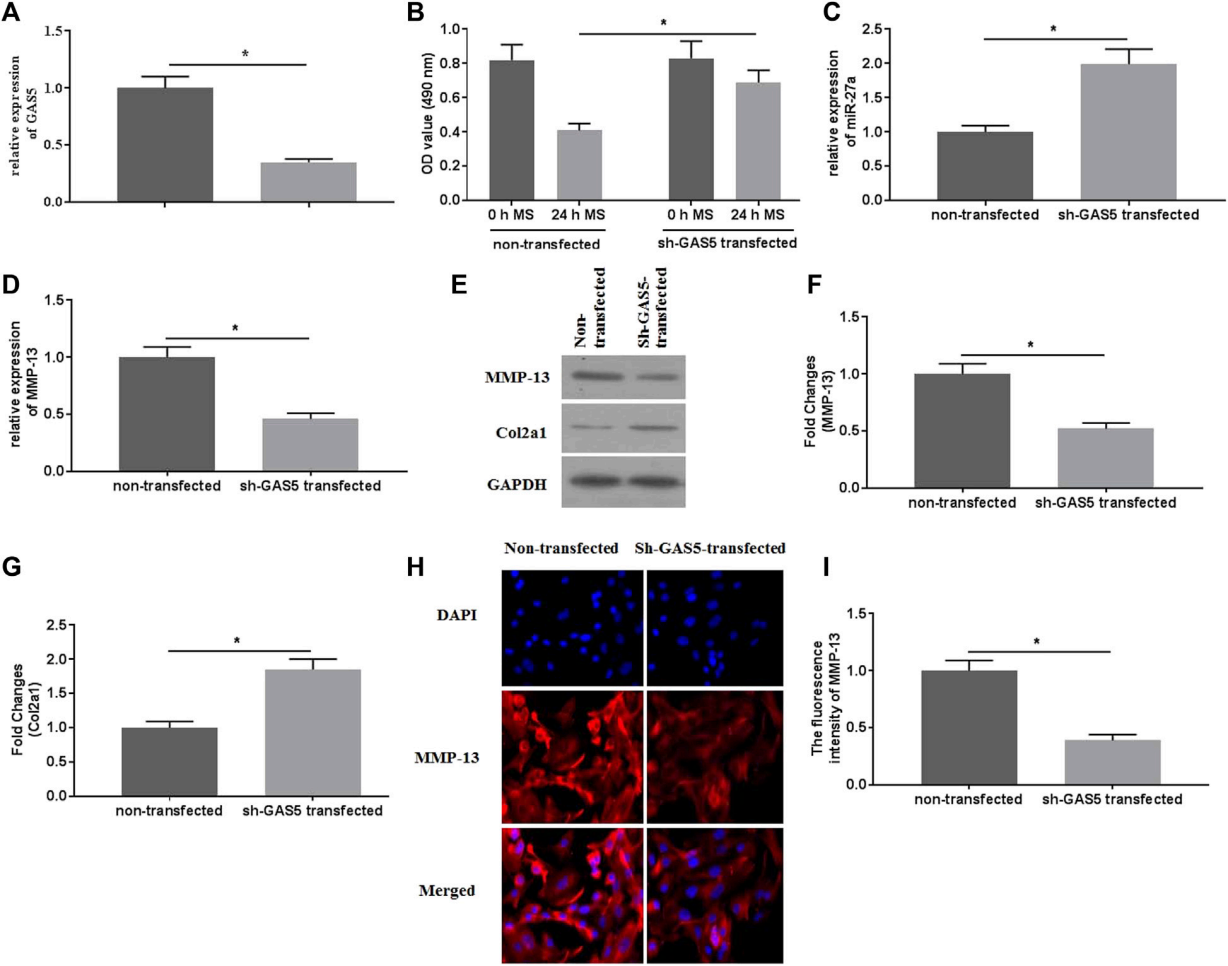
The effects of suppressed GAS5 expression on mechanical stimulation-induced MMP-13 expression were investigated. **(A)** The expression of GAS5 was detected by qRT-PCR in sh-GAS5-transfected chondrocytes. **(B)** The cell viability was determined by CCK-8 assays. **(C)** The expression of miR-27a was determined by qRT-PCR. The mRNA **(D)** expression of MMP-13 was determined by qRT-PCR, and the protein expression of MMP-13 **(E-F)** and Col2a1 **(E** and **G)** were detected bywestern blotting assays. **(H)** The immunofluorescence intensity of MMP-13 was detected. **(I)** The calculation of fluorescence activity of MMP-13. ^*^ *p* < 0.05 and **p < 0.01.

To explore the roles of GAS5 in mechanical stimulation-treated chondrocytes, suppressed expression of GAS5 was conducted by transfection of shRNA-GAS5. The expression of GAS5 was detected by qRT-PCR for identification of successful transfection (Figure 3A). Transfection of shRNA-GAS5 significantly increased the cell viability (Figure 3B), compared with non-transfection. In addition, suppressed expression of GAS5 enhanced the expression of miR-27a (Figure 3C), attenuated the expression of MMP-13 (Figures 3D–F,H,I), and promoted the expression of Col2a1 (Figures 3E,G).

### GAS5 Interacted With miR-27a

**FIGURE 4:**
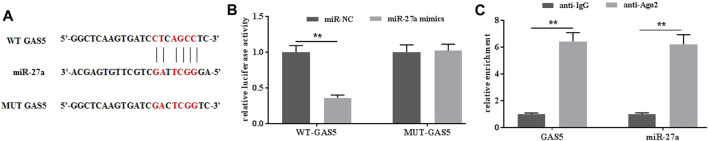
GAS interacted with miR-27a. **(A)** The potential interaction between GAS5 and miR-27a was predicted by Starbase2.0 software. **(B)** The relative luciferase activity was determined in chondrocytes co-transfected with both WT/MUT-GAS5 and miR-27a mimics/miR-NC. **(C)** The interaction between GAS5 and miR-27a was confirmed by RIP assays. **p < 0.01.

To further investigate the possible mechanisms of GAS5 in mediating mechanical stimulation-induced OA development, the potential miRNAs that bind to GAS5 were explored by the predicting software Starbase2.0. It was predicted that miR-27a could be a potential target of GAS5 (Figure 4A). The dual-luciferase reporter assays showed that the luciferase activity in the reporter containing the WT-GAS5 decreased by more than 60%. In contrast, no significant differences were observed in the relative luciferase activities between the NC reporter and the reporter containing the MUT-GAS5 (Figure 4B). In addition, RIP assays demonstrated that GAS5 could physically interact with miR-27a (Figure 4C). Taken together, miR-27a could be a potential target of GAS5.

### Overexpression of miR-27a Attenuated Mechanical Stimulation-Induced MMP-13 Expression

**FIGURE 5:**
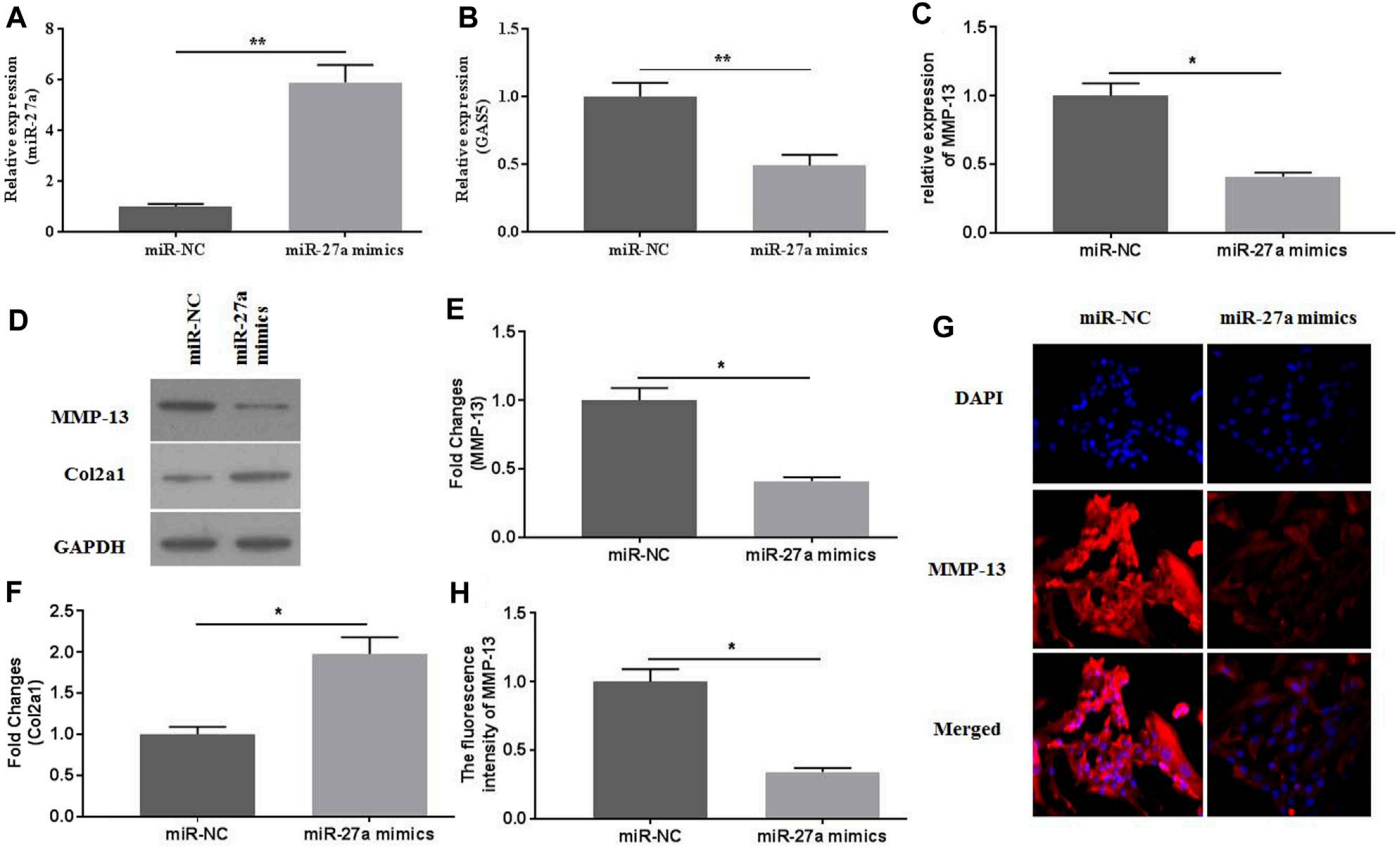
The effects of miR-27a mimic-transfection on mechanical stimulation-induced MMP-13 expression were investigated. The expression of miR-27a **(A)** and GAS5 **(B)** was determined by qRT-PCR in miR-27a mimic-transfected chondrocytes. The mRNA **(C)** expression of MMP-13 was determined by qRT-PCR, and the protein expression of MMP-13 **(D-E)** and Col2a1 **(D** and **F)** were determined by western botting assays. **(G)** The immunofluorescence intensity of MMP-13 was detected. **(H)** The calculation of fluorescence activity of MMP-13. ^*^ *p* < 0.05 and **p < 0.01.

To investigate the roles of miR-27a in mediating mechanical stimulation-induced chondrocytes, miR-27a mimics were transfected into chondrocytes. The expression of miR-27a was detected by qRT-PCR for identification of successful transfection (Figure 5A). It has been reported that MMP-13 was a direct target of miR-27a (Zhang et al., 2020). MiR-27a-mimics transfection effectively decreased the expression of GAS5 (Figure 5B) and MMP-13 (Figures 5C–E, G-H) and increased the expression of Col2a1 (Figures 5D,F). Collectively, mechanical stimulation induced the expression of MMP-13 by mediating the activity GAS5/miR-27a signaling pathway.

### XH Protected Chondrocytes Against Mechanical Stimulation-Induced Damages by Mediating GAS5/miR-27a Signaling Pathway

**FIGURE 6:**
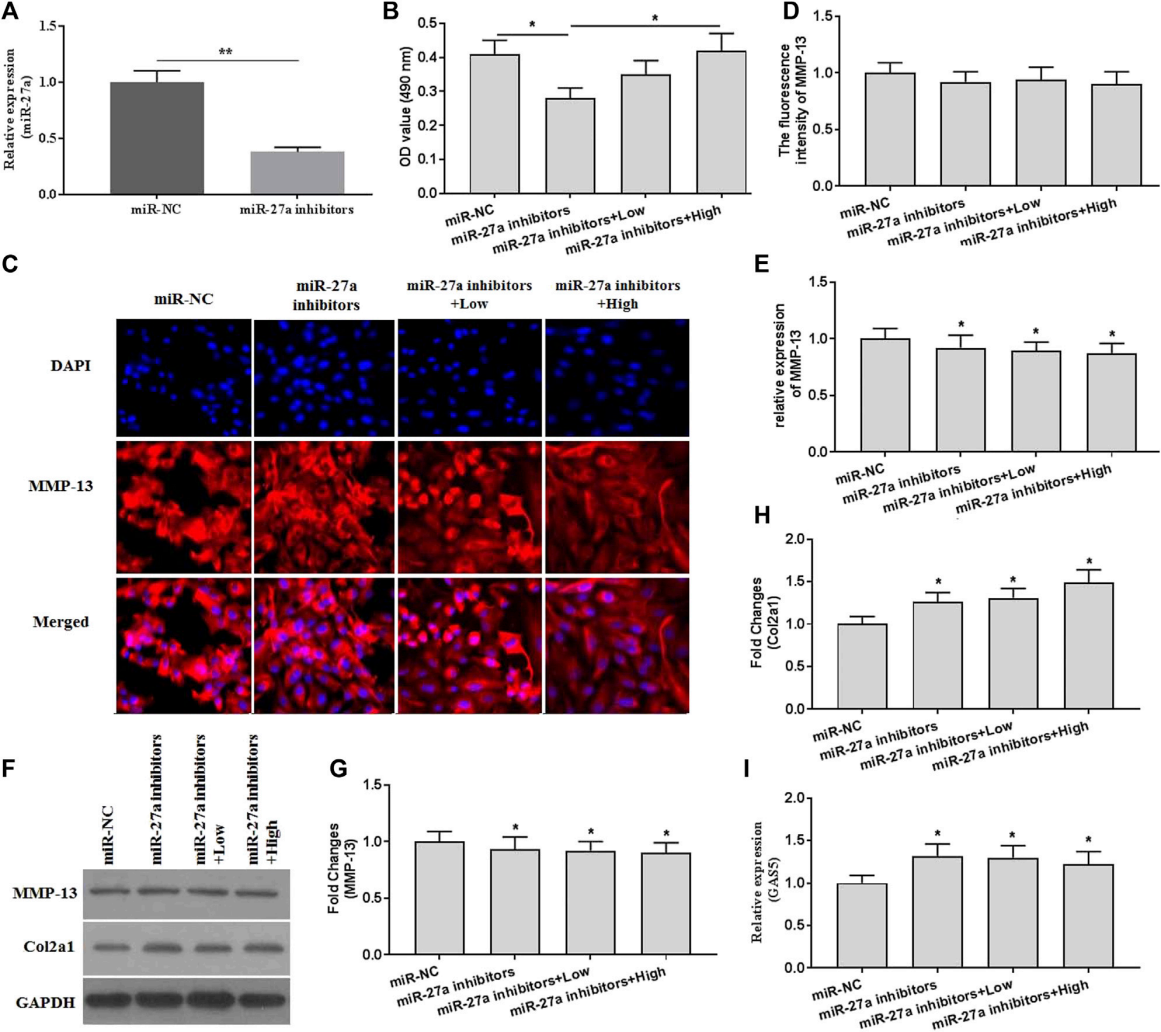
The effects of XH on the expression of MMP-13 in miR-27a inhibitor-transfected chondrocytes with mechanical stimulation were investigated. **(A)** The expression of miR-27a was determined by qRT-PCR in miR-27a inhibitor-transfected chondrocytes. **(B)** The cell viability was determined by CCK-8 assays after mechanical stimulation for 24 h. **(C)** The immunofluorescence intensity of MMP-13 was determined. **(D)** The calculation of fluorescence activity of MMP-13. The mRNA **(E)** expression of MMP-13 was determined by qRT-PCR, and the protein expression of MMP-13 **(F-G)** and Col2a1 **(F** and **H)** were determined by western blotting assays. **(I)** The expression of GAS5 was determined by qRT-PCR in miR-27a inhibitor-transfected chondrocytes. ^*^ *p* < 0.05 and **p < 0.01. NC, negative control; Low, XH (5 μM); High, XH (20 μM).

To investigate whether XH exhibited protective activity against mechanical stimulation-induced damages, miR-27a inhibitors were transfected. The expression of miR-27a was determined by qRT-PCR for identification of successful transfection (Figure 6A). MiR-27a inhibitors transfection abrogated the increased cell viability by XH (Figure 6B). Suppressed expression of miR-27a could reverse the inhibitory effects of XH on MMP-13 and Col2a1 expression, as showed by no significant difference in fluorescence activity (Figures 6C,D) of MMP-13, increased expression of MMP-13 (Figures 6E–G), and decreased expression of Col2a1 (Figures 6F,H). In addition, Suppressed expression of miR-27a up regulated the expression of GAS5 (Figure 6I). Collectively, XH exhibited protective activity against mechanical stimulation-induced MMP-13 by mediating GAS5/miR-27a signaling pathway in chondrocytes.

## Discussion

Abnormal mechanical stimulation associated with obesity, trauma, and joint instability has been demonstrated to change joint loading and be closely related to chondrocytes apoptosis and cartilage degeneration (Guilak, 2011). However, the underlying mechanism of mechanical stimulation in mediating cartilage degeneration is still unclear. It is necessary to explore the roles of mechanical stimulation in OA pathological development. In this article, we found that XH significantly protected against mechanical stimulation-ECM degradation in forced running mice models. *In vitro*, isolated chondrocytes were stimulated with 20% surface elongation at a frequency of 6 cycles/min. XH exhibited protective activity against MMP-13 expression, which could be enhanced by GAS5. Suppressed expression of GAS5 attenuated mechanical stimulation-induced MMP-13 expression. MiR-27a could interact with GAS5, and overexpression of miR-27a significantly decreased the expression of MMP-13. Collectively, XH protected against mechanical stimulation-induced cartilage damages by mediating GAS5/miR-27a signaling pathway.

Articular cartilage is a deficiency of regenerative capacity when subjected to acute or long-term abnormal mechanical stimulation. Under such circumstances, articular cartilage is susceptible to develop degenerative lesions and OA pathology (Fang et al., 2021). Many factors have been demonstrated to contribute to mechanical stimulation-mediated OA development, but their precise roles need to be further elucidated. NF-κB- and MAPK-mediated inflammatory responses are significantly enhanced by overloading (Choi et al., 2019). Wnt/β-catenin signaling is also activated by overloading, leading to up regulation of MMPs activity and degradation of ECM (Pérez-García et al., 2019). Transforming growth factor-β (TGF-β) maintains functions of articular cartilage by transducing Smad2/3 signals, thereby promoting collagen (Col2a1) and fibronectin synthesis and inhibiting ECM degradation induced by overloading (Madej et al., 2016). Mechanical loading also causes differential expression of miRNAs (Kwak et al., 2020) and lncRNAs (He et al., 2021), which regulate the activities of MMPs and the progress of OA development.

Many studies have demonstrated that bulk RNA sequencing and single-cell sequencing data have been used for the analysis in the pathological development of OA (Li et al., 2021). LncRNA LINC00917 and CTD-2246P4.1 have been reported to regulate angiogenesis in OA cartilage by mediating SPHK1 (Chen et al., 2015), which is associated with angiogenesis and promotes the survival of endothelial cells, the processes of cartilage degradation, and the development of OA (Minashima et al., 2014). Increased expression of lncRNA PVT1 in diabetic OA cartilage has been associated with Mankin score and reduced expression of type II collagen by negatively interacting with miR-146a, increasing the productions of inflammatory cytokines, and activating the TGFβ/SMAD4 signaling pathway. LncRNA GAS5 expression is up regulated in OA cartilage tissues. Silence of GAS5 increases the autophagy ability and decreases the apoptosis rate by sponging miR-144 (Ji et al., 2021). Similarly, our study also demonstrated that GAS5 expression was significantly increased by mechanical stimulation *in vivo* and *in vitro*. Suppressed expression of GAS5 effectively ameliorated the expression of MMP-13, which plays a critical role in ECM degradation and OA development.

MicroRNAs suppress genes expression post-transcriptionally. Dysregulation of microRNAs in OA has been reported (Shvedova and Kobayashi, 2020). MiR-29 acts across the development and progression of OA by negatively regulating Smad, NF-κB, and canonical Wnt signaling pathways (Le et al., 2016). MiR-34a/miR-146a/miR-181a has been considered as the potential mediator in mediating the hydrostatic pressure effects on oxidative stress in osteoarthritic chondrocytes. Silencing expression of miR-34a/miR-146a/miR-181a significantly down regulates the expression of MMP-13 and ADAMTS-5 and up regulates the expression of Col2a1, mediating the effects of hydrostatic pressure on chondrocytes apoptosis (Cheleschi et al., 2020). In our study, miR-27a expression was significantly decreased in mechanical stimulation-treated chondrocytes. MMP-13 was a direct target of miR-27a (Zhang et al., 2020), and miR-27a was a direct target of GAS5. Overexpression of miR-27a greatly decreased the expression of MMP-13 and GAS5 and increased the expression of Col2a1. Suppressed expression of miR-27a exhibited reverse effects. In another study, it showed that GAS5 also exaggerates the pathological development of OA by interacting with miR-137 (Gao et al., 2020). Both miR-27a and miR-137 are the downstream factors of GAS5. However, the direct relationship between miR-27a and miR-137 has not been reported. A study showed that miR-137 can ameliorate the cell damages induced by H2O2 (Wang et al., 2021). Consistently, miR-137 is also reported to decrease inflammation, oxidative stress, neuronal injury, and cognitive impairment in mice with ischemic stroke (Tian et al., 2020). Similarly, a study reports that miR-27a mimics can suppress oxygen glucose deprivation (OGD)-induced oxidative injury in HT22 cells (Li et al., 2021). Both miR-137 and miR-27a exhibits protective activities against oxidative stress. However, their relationships needs to be further elucidated.

Recently, natural products have been intensively studied for developing new potential drugs. Harpagoside, isolated from Harpagophytum procumbens, has been demonstrated to protect OA cartilage against degradation by inhibiting the expression of MMP-13 and pro-inflammatory cytokines (Haseeb et al., 2017). Morroniside is a natural compound from Cornus officinalis. It has been reported that morroniside protects against ECM degradation and OA development by decreasing the expression of MMP-3/-13 in the destabilization of the medial meniscus-induced mouse OA models (Park et al., 2021). Alpinetin, a natural flavonoid compound, can effectively reduce TNFα-induced MMP-13 and ADAMTS-5 expression and protect against cartilage destruction in rat OA chondrocytes (Gao et al., 2020). XH has been shown various biological activities, including anti-inflammation, anti-oxidation, anti-proliferation, and anti-angiogenesis (Harikumar et al., 2009; Krajka-Kuźniak et al., 2020). Our previous study shows that XH inhibits the production of inflammatory cytokines by activating the NRF2/HO-1 signaling pathway, protecting OA chondrocytes (Zhang et al., 2021). In this study, XH exhibited inhibitory activity against ECM degradation by mediating GAS5/miR-27a signaling pathway *in vivo* and *in vitro*.

Interestingly, how XH mediated GAS5/miR-27a signaling pathway is still unclear. The biological activity of XH has been demonstrated by activating the NRF2/HO-1 signaling, and whether NRF2/HO-1 signaling is involved in the mechanism of XH in mediating the GAS5/miR-27a pathway still needed to be elucidated. GAS5 has been reported to enhance oxidative stress in melanoma cells (Xu et al., 2020), macrophages (Zhang et al., 2021), and rats with cerebral ischemic stroke (Wu et al., 2021). MiR-27a expression can be activated by NRF2/HO-1 signaling in DSS-induced colitis mouse model (Dun et al., 2021). It is plausible that activation of NRF2/HO-1 signaling might suppress the expression of GAS5/miR-27a axis, which should be confirmed in chondrocytes. And this might be involved in the pharmacological activity of XH in protecting against OA development.

There are limitations to this study. CMC is often used as a good cosolvent to improve the bioavailability of natural products. Recently, CMC has been demonstrated to reduce post-prandial plasma glucose levels (Miehle et al., 2021). No control group with CMC administration was designed, and this made the possibility to confuse the biological activity of XH alone or combined with CMC. It needed more efforts to elucidate the possible roles of CMC in the biological activity of XH *in vivo*.

## Conclusion

XH protected against mechanical stimulation-induced ECM degradation in OA chondrocytes by mediating the GAS5/miR-27a/MMP-13 signaling pathway.

## Data Availability

The original contributions presented in the study are included in the article/supplementary material, further inquiries can be directed to the corresponding authors.
